# Loop A Is Critical for the Functional Interaction of Two *Beta vulgaris* PIP Aquaporins

**DOI:** 10.1371/journal.pone.0057993

**Published:** 2013-03-04

**Authors:** Cintia Jozefkowicz, Pablo Rosi, Lorena Sigaut, Gabriela Soto, Lía Isabel Pietrasanta, Gabriela Amodeo, Karina Alleva

**Affiliations:** 1 Departamento de Biodiversidad y Biología Experimental, Facultad de Ciencias Exactas y Naturales, Universidad de Buenos Aires, Buenos Aires, Argentina; 2 Instituto de Química Física de los Materiales, Medio Ambiente y Energía (INQUIMAE-CONICET), Facultad de Ciencias Exactas y Naturales, Universidad de Buenos Aires, Buenos Aires, Argentina; 3 Centro de Microscopías Avanzadas (CMA) y Departamento de Física, Facultad de Ciencias Exactas y Naturales, Universidad de Buenos Aires, Buenos Aires, Argentina; 4 Instituto de Genética “Ewald A. Favret”, CICVyA, INTA, Castelar, Argentina; 5 Consejo Nacional de Investigaciones Científicas y Técnicas (CONICET), Buenos Aires, Argentina; University of Bern, Switzerland

## Abstract

Research done in the last years strongly support the hypothesis that PIP aquaporin can form heterooligomeric assemblies, specially combining PIP2 monomers with PIP1 monomers. Nevertheless, the structural elements involved in the ruling of homo *versus* heterooligomeric organization are not completely elucidated. In this work we unveil some features of monomer-monomer interaction in *Beta vulgaris* PIP aquaporins. Our results show that while *Bv*PIP2;2 is able to interact with *Bv*PIP1;1, *Bv*PIP2;1 shows no functional interaction. The lack of functional interaction between *Bv*PIP2;1 and *Bv*PIP1;1 was further corroborated by dose-response curves of water permeability due to aquaporin activity exposed to different acidic conditions. We also found that *Bv*PIP2;1 is unable to translocate *Bv*PIP1;1-ECFP from an intracellular position to the plasma membrane when co-expressed, as *Bv*PIP2;2 does. Moreover we postulate that the first extracellular loop (loop A) of *Bv*PIP2;1, could be relevant for the functional interaction with *Bv*PIP1;1. Thus, we investigate *Bv*PIP2;1 loop A at an atomic level by Molecular Dynamics Simulation (MDS) and by direct mutagenesis. We found that, within the tetramer, each loop A presents a dissimilar behavior. Besides, *Bv*PIP2;1 loop A mutants restore functional interaction with *Bv*PIP1;1. This work is a contribution to unravel how PIP2 and PIP1 interact to form functional heterooligomeric assemblies. We postulate that *Bv*PIP2;1 loop A is relevant for the lack of functional interaction with *Bv*PIP1;1 and that the monomer composition of PIP assemblies determines their functional properties.

## Introduction

Plant water channels are a huge family of proteins; among them, PIP (*p*lasma membrane *i*ntrinsic *p*roteins) aquaporins are important water transporters. Traditionally, it is considered that PIP aquaporins cluster in two groups: PIP1 and PIP2, but recently it was reported that the common ancestor of mono and dicot plant aquaporins could have three types of PIPs that originated three PIP-like clusters instead of two: i- PIP Cluster I (PIPCLI), which corresponds to the classical PIP1 group, ii- PIP Cluster II (PIPCLII) and iii- PIP Cluster III (PIPCLIII), these last two correspond to most PIP2 aquaporins [1]. Regardless the phylogenetic organization, it is considered that all aquaporins are tetramers [2]–[5]. Notwithstanding, it was demonstrated that each monomer in the tetramer is a functional unit [6], [7].

Likewise, as other multimeric proteins, aquaporins oligomers composition could be homooligomeric or heterooligomeric. Among mammal aquaporins, AQP1 was reported to be homotetrameric [8] while AQP4 exists in two splicing isoforms AQP4M1 (starting at Met1) and AQP4M23 (starting at Met23), which could assemble in the plasma membrane as homo or heterotetramers [9] and are also able to be organized in orthogonal arrays of particles (OAPs) [10]. Regarding plant aquaporins, major research about tetramer composition has been performed for the PIP family. Experimental evidence points that PIP aquaporins can be organized as heterooligomers under particular, not yet fully determined, circumstances. Although the physical and functional interaction among different PIPs has been probed, it is still not elucidated if the protein complex formed is heterooligomeric (homotetramers of different PIPs in contact) or heterotretrameric (different PIP monomers organized in a single tetramer). The functional interaction was mainly studied by means of co-injection of PIP cRNA in *Xenopus laevis* oocytes and many results show that an interaction between different PIPs occurs. For instance, this interaction was reported among maize PIPs, for *Zm*PIP1;2 with several *Zm*PIP2, for tobacco *Nt*PIP1;1 with *Nt*PIP2;1, for grape berry *Vv*PIP1;1 with *Vv*PIP2;2 and for wheat *Td*PIP1;1 with *Td*PIP2;1 confirming that this behavior occurs among all PIP groups [11]–[15]. Moreover, functional and physical interaction was reported between members of a same group [11], [16].

Many relevant evidences about PIP interaction can be mentioned. First, a positive cooperation resulting in an increase in the osmotic water permeability (P*_f_*) of oocyte plasma membrane is seen when *Zm*PIP2s are co-expressed with *Zm*PIP1;2 [11]. Moreover it was shown for *Zea mays* PIPs that PIP1 are retained in the endoplasmic reticulum (ER) while PIP2 are targeted to the plasma membrane, but they are co-localized in the plasma membrane, as result of their interaction, when co-expressed in the same cell [13]. A second evidence is the modification of pH sensing of oocytes co-expressing *Bv*PIP2;2 and *Bv*PIP1;1 in comparison with oocytes expressing *Bv*PIP2;2 alone [17]. Also co-expression of tobacco PIPs, *Nt*AQP1 and *Nt*PIP2;1, studied by means of bimolecular fluorescence complementation (BiFC) experiments, size exclusion chromatography and gel electrophoresis points to the formation of heterotetramers; furthermore functional analysis of artificial tobacco PIP tetramers with a defined proportion of *Nt*AQP1 and *Nt*PIP2;1 indicate that membrane permeability was modified by tetramer composition [18]. Finally, a recent work states that the conformational arrangement of maize PIP2 monomers in PIP1-PIP2 heterooligomers is different from that in PIP2 homooligomers [19].

It is interesting to remark that PIPs have a great evolutionary constraint in comparison with other plant aquaporin subfamilies, this high evolutionary constraint may be due to functional constraint [1]. It has been described that proteins that are part of complexes tend to evolve at a relatively slow rate in order to improve the co-evolution with their interacting partners [20]. The high evolutionary constraint found for PIPs can be related to the above-mentioned physical interaction that occurs among different members of the subfamily and modulate their activity.

Among the biological phenomena regulating protein function, control of homooligomerization *vs* heterooligomerization is an important one. In this context, PIP oligomerization is still emerging as an area of investigation. Aquaporin tetramerization must be studied as a crosstalk mechanism between extracellular signals and water or solute transport, as response.

Here, we focused our study in *Beta vulgaris Bv*PIP2;1 which seems to be unable to functionally interact with *Bv*PIP1;1. *Bv*PIPs were studied in terms of functional interaction by means of cRNA co-injection in *Xenopus laevis* oocytes, pH inhibition experiments and confocal fluorescent microscopy. Furthermore, on the basis of differences found in *Bv*PIP2;1 primary sequence at the first extracellular loop when compared with the same loop of most PIP2, we investigate *Bv*PIP2;1 loop A by direct mutagenesis. We also characterized *Bv*PIP2;1 loop A at an atomic level by Molecular Dynamics Simulation (MDS). From the results obtained in this work we proposed that *Bv*PIP2;1 loop A is involved in the failure of this aquaporin to interact with *Bv*PIP1;1.

We believe this work is a contribution to unravel how PIPs interact to form functional heterooligomeric assemblies.

## Materials and Methods

### Plasmid Constructions

The coding regions of *Bv*PIP1;1, *Bv*PIP2;1 and *Bv*PIP2;2 (GenBank sequences GQ227845.1, U60148.1 and GQ227846.1, respectively) and the mutants (*Bv*PIP2;1N64H/E65Q and *Bv*PIP2;1N64I/E65Q) were cloned into the BglII and SpeI sites of a pT7Ts derived vector containing T7 RNA polymerase promoter and carrying 5′- and 3′- translated region of the *Xenopus laevis* β-globin gene for enhanced expression [17]. Monomeric fluorescent protein ECFP was fused to the C-terminal of *Bv*PIP1;1 and EYFP was fused to the C-terminal of *Bv*PIP2;2. Then they were sub-cloned into the pT7Ts-compatible Xenopus expression vector by PCR. All constructs were confirmed by DNA sequencing (Macrogen Inc, USA).

### Phylogenetic Analyses

Phylogenetic and molecular evolutionary analyses were performed as detailed in [1]. Briefly, the analyses were conducted by using MEGA version 4.0 [21]; history reconstruction of PIPs was restricted to protein sequences with high amino acid identity (>25%). Phylogenetic trees were constructed using the neighbor-joining (NJ) method with genetic distances computed using Poisson correction model. This analysis was developed by setting the following parameters: substitutions to include = all, gaps/missing data = pair wise deletion, phylogeny test = bootstrap 500 replicates and root on midpoint.

### Sequence Analysis

Sequence search was performed using BLASTP tool (http://blast.ncbi.nlm.nih.gov/Blast.cgi). Only the following sequences, with reported interaction with PIP1, were selected: *At*PIP2;3 (*Arabidopsis thaliana* GI: 15228096), *Bv*PIP2;2 (*Beta vulgaris* GI: 1402833), *Mp*PIP2;1 (*Mimosa pudica* GI: 60498684), *Nt*PIP2;1 (*Nicotiana Tabacum* GI: 17017257), *Os*PIP2;2 (*Oryza sativa* GI: 75291011), *Os*PIP2;4 (*Oryza sativa* GI: 75299345), *Tt*PIP2;1 (*Triticum turgidum* GI: 158324048), *Vv*PIP2;2 (*Vitis vinifera* GI: 124702519), *Zm*PIP2;1 (*Zea mays* GI: 29650727), *Zm*PIP2;4 (*Zea mays* GI: 13447807), *Zm*PIP2;5 (*Zea mays* GI: 29650729).

Multiple sequence alignment were performed with ClustalW version 2 [22] and retrieved to WebLogo (http://weblogo.berkeley.edu/logo.cgi) to generate a graphical representation of the patterns within the multiple sequence alignment [23], [24].

### Mutations of *Bv*PIP2;1 cDNA

Mutated cDNA encoding *Bv*PIP2;1 N64H/E65Q and *Bv*PIP2;1 N64I/E65Q were obtained by site direct mutagenesis (Quickchange, Stratagene, USA) following the manufacter’s recommendations using custom-made oligonucleotides primers (Eurofins MWG Operon, USA). 5′-CAGTTGCAACTGTTATTGGTTACAAACATCAAACTGACCCTTGTGC-3′ and 5′-GCACAAGGGTCAGTTTGATGTTTGTAACCAATAACAGTTGCAACTG-3′ were used to mutate the asparagine at position 64 (N64H) and glutamic acid 65 (E65Q), respectively to histidine and glutamine. Also the custom-made oligonucleotides (Eurofins MWG Operon, USA) 5′-CAGTTGCAACTGTTATTGGTTACAAAATTCAAACTGACCCTTGTGC-3′ and 5′-GCACAAGGGTCAGTTTGAATTTTGTAACCAATAACAGTTGCAACTG-3′ were used to mutate the asparagine at position 64 (N64I) and glutamic acid 65 (E65Q), respectively, to isoleucine and glutamine. DNA sequencing (3730xl DNA analyzer Macrogen Inc. Seul, Korea) was used to corroborate all mutations.

### *In Vitro* RNA Synthesis

The capped complementary RNAs (cRNA) encoding *Bv*PIP1;1 and *Bv*PIP2;2 were synthesized *in vitro* using the *mMESSAGE mMACHINE T7 High Yield Capped RNA Transcription Kit* (Ambion, Austin, Texas, USA) as described previously [17]. cRNA encoding *Bv*PIP2;1 and its mutants were synthesized *in vitro* with *mMESSAGE mMACHINE T7 Ultra Kit* (Ambion, USA) by using anti reversed cap analog (ARCA) and poly (A) tailing reagents as previously detailed in [17]. The synthesized products were suspended at a final concentration of 0,1 µg µL^−1^ in RNAse-free water supplemented with Recombinant RNasin (Ribonuclease inhibitor, Promega, USA) and stored at −20°C until used [25]. The cRNA was quantified by fluorescence using Quant-iT RNA Assay Kit (Invitrogen, UK). Agarose gel electrophoresis and GelRed (BioAmerica Biotech Inc., USA) staining were used to check the absence of unincorporated nucleotides in the cRNA. At least four independent cRNA syntheses were assayed. Results from experiments performed with different oocytes batches were not pooled; therefore all the experiments shown in this work are representative for at least three different experiments.

Before injecting, cRNA was diluted in order to inject a proper amount per oocyte. Then, all masses of cRNA injected in the same experiment were considered as relative to an arbitrary unit of measure. For example, if (1) is 1,25 ng of cRNA injected per oocyte, (3) is three times this quantity.

### Oocyte Water Transport Assays

Osmotic water permeability (P*_f_*) of oocytes injected with cRNA or non-injected, was determined by measuring the rate of oocyte swelling as explained before [26]. Briefly, osmotic water permeability (P*_f_*) was determined by measuring the rate of oocyte swelling induced by a hypo-osmotic shock of 160 mOsm kg^−1^ H_2_O. Changes in cell volume were video-monitored by a VX-6000 color video-camera (Microsoft, CA, USA) attached to a zoom stereo-microscope (Olympus SZ40, Olympus Co., Japan). The cell swelling was video-captured in still images (each 10 s during 70 s) using the AMCaP version 9.20 (http://noeld.com/programs.asp?cat¼video#AMCap) and then the images were analyzed by treating each oocyte image as a growing sphere whose volume could be inferred from its cross-sectional area (software *Image J* version 1.37, http://rsb.info.nih.gov/ij/).

The osmotic water permeability (P*_f_*) was calculated according to [27] and [28]. Non-injected oocytes were used as negative controls because no significant differences were found between this condition and water injected oocytes. All osmolarities were determined using a vapor pressure osmometer (5600C Wescor Inc. USA).

### pH Inhibition Assays

For pH inhibition experiments the oocyte internal (cytosolic) pH was modified following an already described protocol in [29] and the modifications made in [17]. Briefly, the oocyte internal pH was acidified by pre-incubating them for 15 min in different pH solutions (NaAc Solution: 50 mM NaAc, 20 mM MES for 5.8–6.7 pH interval or HEPES for the 7.0–7.6 pH interval), supplemented with mannitol 1 M until the desired osmolarity was achieved (∼200 mOsmol kg^−1^ H_2_O). To calculate final oocyte intracellular pH a calibration curve described previously in our laboratory was used [17]. The swelling response was performed by transferring the oocyte to the same solution diluted 5-fold with distilled water in order to induce the osmotic shock. In all treatments, negative controls were performed by submitting non-injected oocytes to the same protocol and the percentage of inhibition was calculated using the formula: Inh (%) = [1−(P*_f_*_pH 6.4_– P*_f_*_NI_)/(P*_f_*_pH 7.0_– P*_f_*_NI_)] 100, where NI states non-injected. Data were fitted to a sigmoidal dose-response curve P*_f_* = P*_f_* max*pH_int_^h^/(EC50^h^+pH_int_^h^)+P*_f_* min, using Graph Pad Prism (version 3.02).

### Assessment of Fluorescent PIP Proteins Expression in Oocyte Plasma Membrane

Confocal fluorescence microscopy was used to localize the respective PIP isoforms tagged with ECFP or EYFP in *Xenopus laevis* oocytes. As a marker of the interior of the oocyte we used tetrametylrhodamine (TMR) dextran (10,000 MW; Invitrogen-Molecular Probes, USA) an unconjugated non-specific fluorochrome marker that stays in the area of the cortical granules and allows distinguishing plasma membrane from cytosol [30]. Briefly, 3–4 days after cRNA injection and 40 minutes prior to imaging, oocytes were microinjected with 50 nl of a 33 mM aqueous solution of TMR-dextran.

Fluorescence images of ECFP or EYFP distribution together with TMR were obtained with a FluoView1000 spectral confocal scanning microscope (Olympus Co., Japan), employing a 60X UPlanSapo oil immersion objective lens NA1.35. In order to avoid crosstalk, images were recorded line by line in a sequential order. In the case of ECFP and TMR, they were excited using the 458 nm and 515 nm line of a multiline Argon laser and the emitted fluorescence was detected in the 475–500 nm and 555–655 nm range, respectively. When EYFP and TMR were employed, 488 nm line of the Argon laser and 543 nm He-Ne were used and the emitted fluorescence was detected in the 500–535 nm and 570–670 nm range, respectively. Except for z-stacks, images were obtained using Kalman filtering.

Autofluorescence (monitored in control oocytes) was negligible in comparison to cells expressing the fluorescent PIP. We analyzed 3–4 oocytes for each condition from at least 7 donor frogs.

For reconstructing 3D views, confocal (x–y) images were collected at various focal depths into the oocyte with 100 nm steps. The stack of images was deconvoluted employing the classic maximum likelihood estimation as a restoration method, then iso-sampled and surface rendered for 3D visualization employing Huygens Professional Software (Scientific Volume Imaging, Hilversum, The Netherlands).

### Statistics

Results are reported in the form of means ± SEM. Significant differences between treatments were calculated using the Student’s t-test.

### Homology Modeling (HM)

In order to explore the structure and dynamics of loop A we built an homology model (HM) of *Bv*PIP2;1 sequence. The sequence was submitted to the Swiss-Model [31] public server and the model was stirred to superimpose over the chain A of the crystal of the *So*PIP2;1 (2B5F pdb code) [5]. The sequence identity calculated was 86.6% and the retrieved model was very well superimposed over the chain A, especially in the alpha helix regions. In order to explore the homotetramer geometry, four identical models were superimposed over each monomer of the crystal structure.

### Molecular Dynamics Simulation (MDS) Methodology

In order to characterize *Bv*PIP2;1 loop A dynamics, we built the topology file based on the homotetramer that was fully solvated in an octahedral box of TIP3P water model with periodic boundary conditions. The MDS was performed using AMBER 11 [32]. The model was prepared with a 3-steps protocol. First, we ran 2000 steps of side chains optimization, second a 50000 steps of thermalization at 300 K with a Berendsen thermostat at constant volume, and third, a 50000 steps at the same temperature with constant pressure (isotropic position scaling) of 1 atm. Finally, 30 ns of MDS under the NPT ensemble condition were performed. This system remained stably structured along the whole MDS.

## Results

### PIP1-PIP2 Interaction

Three PIP aquaporins from *Beta vulgaris* are known, *Bv*PIP2;1 (GenBank: U60148.1, GI:1402834) (corresponding to CLIII; Figure S1), *Bv*PIP2;2 (GenBank: U60147.1, GI:1402832) (corresponding to CLII; Figure S1), and *Bv*PIP1;1 (GenBank: U60149.1, GI:1402836) (corresponding to CLI; Figure S2). Previously, it was shown that *Bv*PIP2;2 is able to functionally interact with *Bv*PIP1;1 [17].

**Figure 1:**
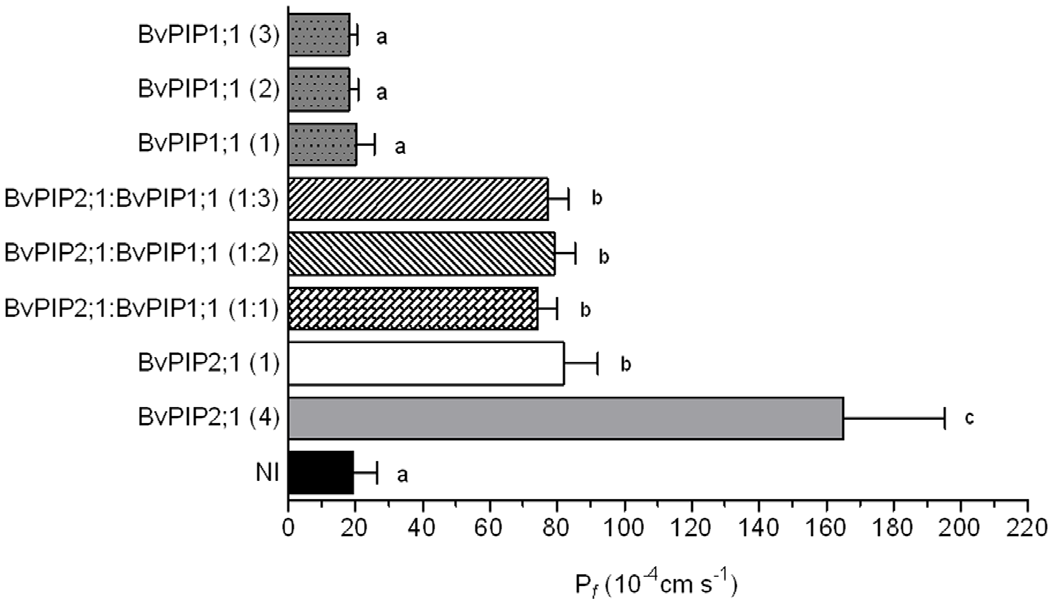
Effect of *Bv*PIP2;1 and *Bv*PIP1;1 co-expression on oocyte plasma membrane permeability (P*_f_*). Different amounts of cRNA of *Bv*PIP2;1, *Bv*PIP1;1 or a mix of *Bv*PIP2;1 and *Bv*PIP1;1 (*Bv*PIP2;1:*Bv*PIP1;1) were injected in *Xenopus* oocytes and after three days osmotic water permeability coefficient (P*_f_*) was determined. In brackets is the relative quantity of cRNA injected in each oocyte, being 1 equal to 0,3 ng, and 2 or 3, two or three fold that amount, respectively. A four-fold injection of *Bv*PIP2;1 (4) was used as a control to show that the expression system was not saturated, NI are non-injected oocytes. Data are expressed as mean values (mean P*_f_* ±SEM, n = 12−15). The figure shows representative data from five independent experiments. Different letters indicate significance between bars (p<0.05). All P*_f_* corresponding to oocytes co-injected with different cRNA ratios of *Bv*PIP2;1: *Bv*PIP1;1 were not significantly different from P*_f_* of *Bv*PIP2;1 (1) injected oocytes.

Here, we focused on the functional features of *Bv*PIP2;1. Figure 1 shows that the expression of *Bv*PIP2;1 in *Xenopus laevis* oocytes lead an increase in plasma membrane osmotic water permeability coefficient compatible with an active water channel. Oocyte co-expressing *Bv*PIP2;1 with *Bv*PIP1;1 show not significantly different P*_f_* values in comparison with *Bv*PIP2;1 expressed alone, even at different cRNA ratios of *Bv*PIP2;1:*Bv*PIP1;1 injected. Therefore, *Bv*PIP2;1 is an active aquaporin which is unable to functionally interact with *Bv*PIP1;1.

**Figure 2:**
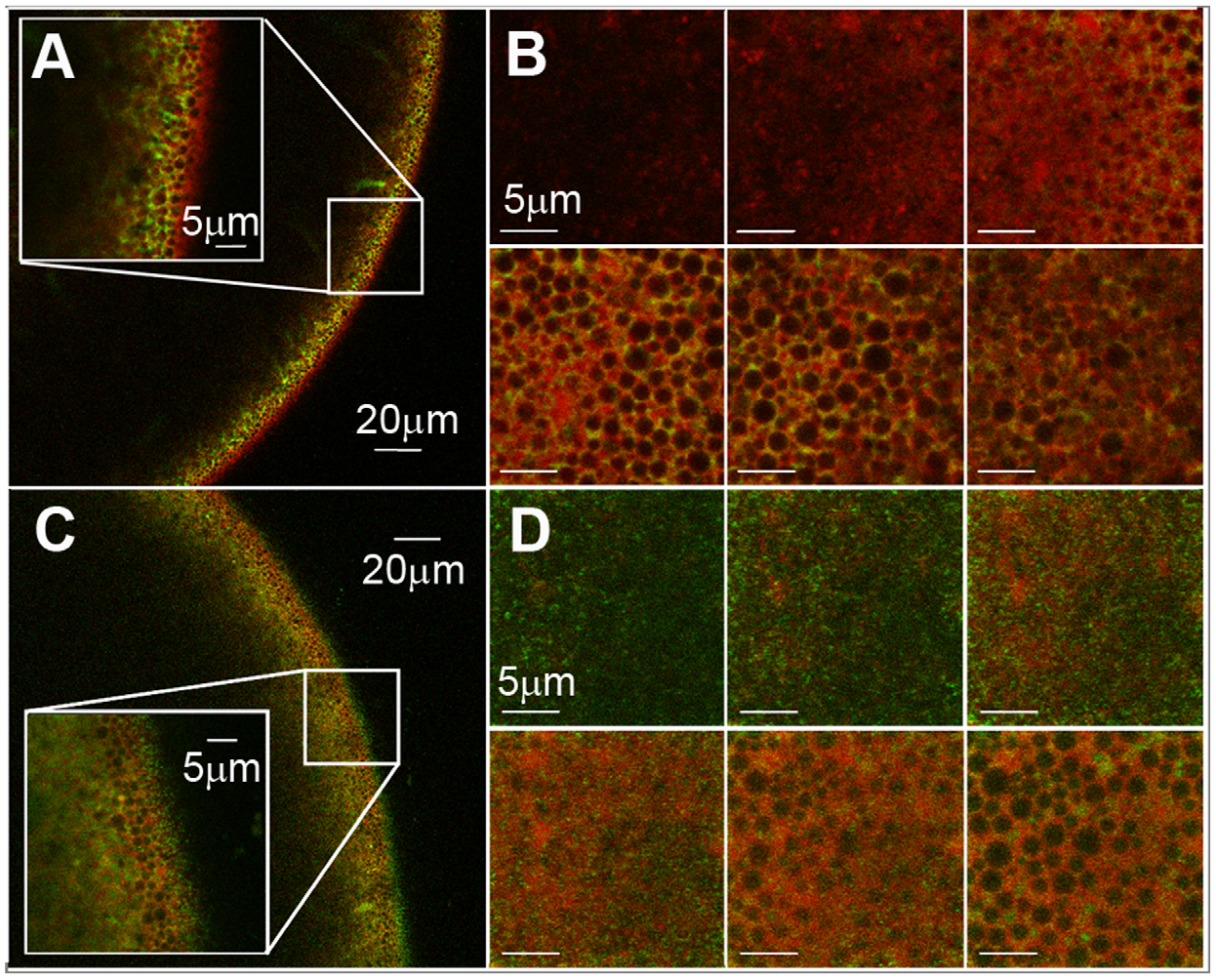
Localization of *Bv*PIP1;1-ECFP and *Bv*PIP2;2-EYFP in *Xenopus laevis* oocytes. Radial (x–z) confocal images of *X*. *laevis* oocytes expressing *Bv*PIP1;1-ECFP (A) (green) and *Bv*PIP2;2- EYFP (C) (green), previously injected with TMR-Dextran (red). The oocyte surface is on the right of each image frame and the interior of the oocyte is to the left. Inside each image the enlargement of the indicated square section is shown. Confocal (x–y) images collected at various focal depths into the *X*. *laevis* oocyte expressing *Bv*PIP1;1-ECFP (B) and *Bv*PIP2;2-EYFP (D) at 1µm steps from outside the oocyte till the cortical granules level, approximately 5 µm from the plasma membrane, are shown.

In order to correlate the previous water transport parameters of co-expressing systems (*Bv*PIP2;1-*Bv*PIP1;1) with the cellular localization of *Bv*PIP1;1, we designed the following fusion proteins: *Bv*PIP2;2-EYFP (as a control of plasma membrane localization) and *Bv*PIP1;1-ECFP. When these fusion proteins are injected in oocytes, the analysis made by confocal fluorescence microscopy show that the fluorescent signal of *Bv*PIP1;1-ECFP is localized in the same area of TMR-dextran (marker of the interior of the cell) (Figure 2 A and B) and that fluorescence due to *Bv*PIP2;2-EYFP (our control for plasma membrane localization) is found in the limit of the cell (Figure 2 C and D). This result indicates that *Bv*PIP1;1-ECFP is retained in the interior of the cell while *Bv*PIP2;2-EYFP is located at the plasma membrane. The localization of *Bv*PIP2;2-EYFP is consistent with the high P*_f_* reported for oocytes injected with this aquaporin [17]. On the other hand, when cRNA of *Bv*PIP1;1 is injected in oocytes, P*_f_* is not different from non-injected oocytes (as shown in Figure 1); this is in accordance with the lack of *Bv*PIP1;1-ECFP in oocytes plasma membrane.

**Figure 3:**
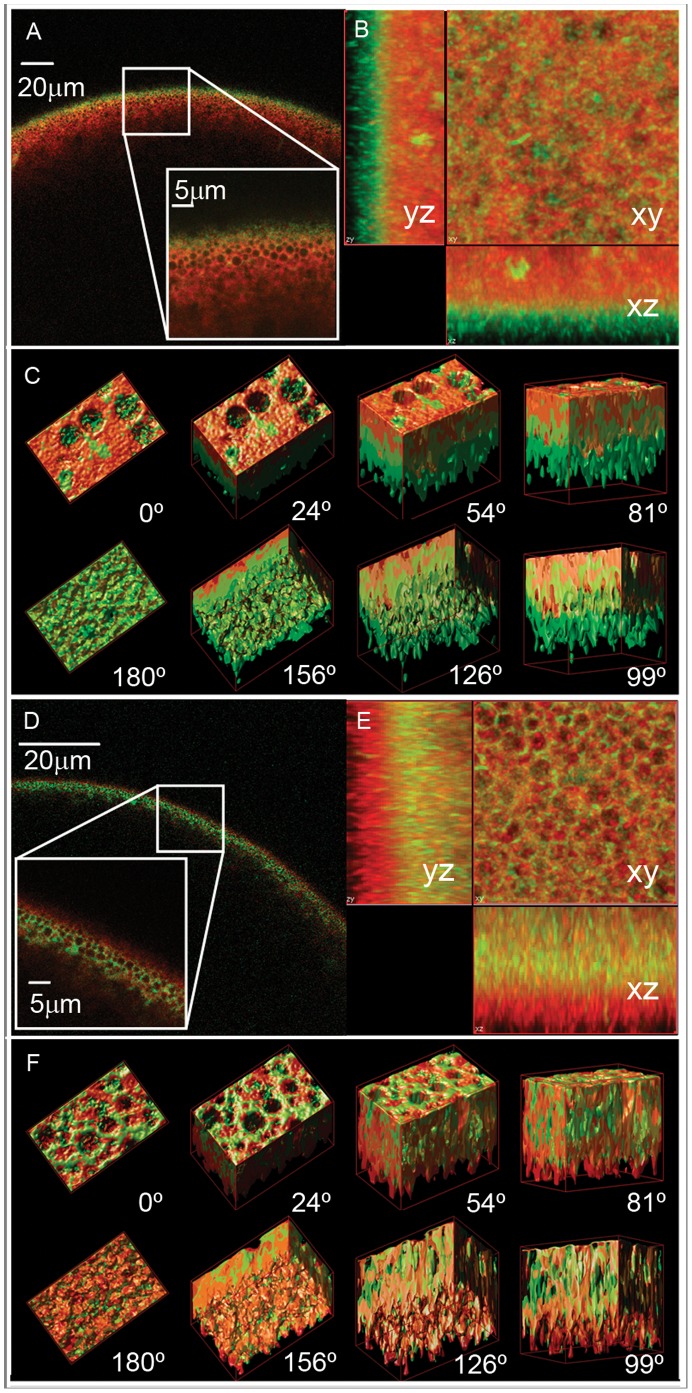
Localization of *Bv*PIP1;1-ECFP when co-expressed with *Bv*PIP2;2 or *Bv*PIP2;1 in *Xenopus laevis* oocytes. Radial (x–z) confocal images of *X*. *laevis* oocytes co-expressing *Bv*PIP1;1-ECFP:*Bv*PIP2;2 (green) (A) or co-expressing *Bv*PIP1;1-ECFP:*Bv*PIP2;1 (green) (D), both previously injected with TMR-Dextran (red). The oocyte surface is near the top of each image frame and the interior of the oocyte is in the bottom. Inside the image the enlargement of the indicated square section is shown. Stack of confocal (x–y) images were collected at various focal depths into the oocyte and then deconvolved and surface-render reconstructed with Huygens Professional Software. (B) Projections of the z-stack of images acquired with 100 nm step for oocytes co-expressing *Bv*PIP1;1-ECFP:*Bv*PIP2;2 (green) and (E) for oocytes co-expressing *Bv*PIP1;1-ECFP:*Bv*PIP2;1(green). (C) and (F) shows several views of the 3D reconstructed images for oocytes co-expressing *Bv*PIP1;1-ECFP:*Bv*PIP2;2 (green) and *Bv*PIP1;1-ECFP:*Bv*PIP2;1(green), respectively. The 0° view corresponds to the cortical granules level inside the oocyte and 180° to the plasma membrane plane (approximately 5 µm from the cortical granules level).

Knowing that *Bv*PIP1;1-ECFP is retained in the interior of the cell, we proceed to test its localization when co-expressed with *Bv*PIP2;1 and *Bv*PIP2;2. Interestingly, when the oocytes are co-injected with cRNAs of *Bv*PIP1;1-ECFP and *Bv*PIP2;2, the fluorescence signal is mainly located at the plasma membrane (Figure 3 A, B and C). However, fluorescent signal is still located at the interior of the oocyte when *Bv*PIP1;1-ECFP is co-expressed with *Bv*PIP2;1 (Figure 3 D, E and F). This result shows that the localization of *Bv*PIP1;1-ECFP is modified from the interior of the oocyte to the plasma membrane only in the presence of *Bv*PIP2;2, suggesting that *Bv*PIP2;1 does not promote the trafficking of *Bv*PIP1;1-ECFP to the plasma membrane. Again, the localization of *Bv*PIPs is in accordance with the functional results shown in Figure 1.

The P*_f_* values of oocytes expressing fluorescent-tagged PIPs are in accordance with the data obtained for the corresponding wild type aquaporins corroborating that the tags are not affecting aquaporin activity (Figure S3).

### *Bv*PIP2;1 pH Response

It is well known that most PIPs are inhibited by cytosolic acidification. The gating mechanism was explained by the protonation of a conserved histidine residue located on the intracellular loop D [29]. In the case of *Bv*PIP2;1 the conserved histidine responsible of cytosolic pH sensing seems to be H193 regarding its alignment with others PIP2.

**Figure 4:**
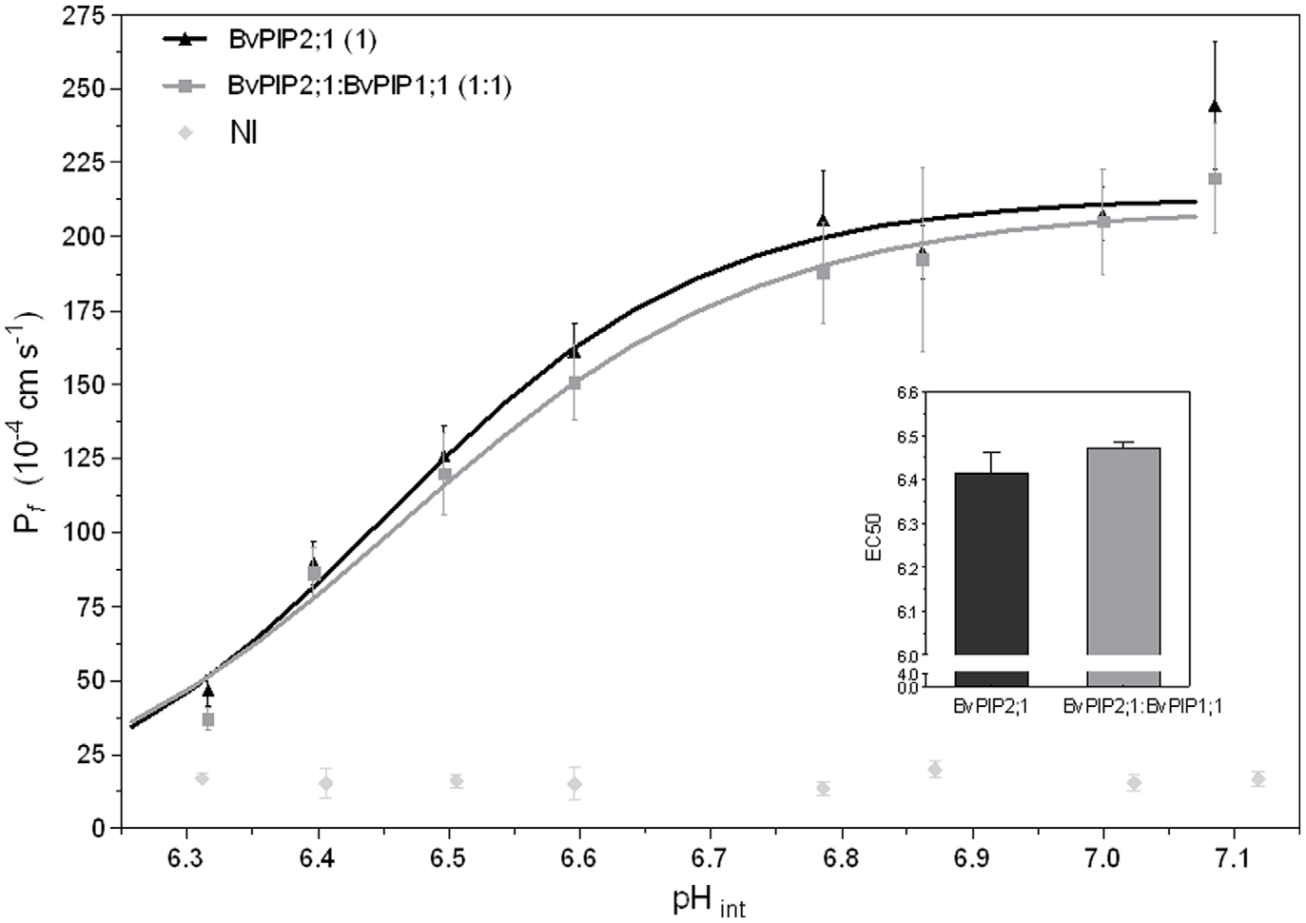
Effect of pH on oocytes membrane P*_f_* expressing *Bv*PIP2;1 alone or co-expressing *Bv*PIP2;1 with *Bv*PIP1;1. To evaluate cytosolic pH sensing, oocytes expressing *Bv*PIP2;1, co-expressing *Bv*PIP2;1-*Bv*PIP1;1 in a 1∶1 mass ratio or non-injected (NI) were incubated 15 min at different pH media. Then each oocyte was transferred to a fivefold-diluted medium at the same pH and the swelling assay was performed as described in Materials and Methods. Three independent experiments were performed and for each pH condition 7–10 oocytes were tested. The main figure shows representative values obtained on a same batch of oocytes (mean P*_f_* ±SEM). Data were fitted to a sigmoidal dose-response curve using Graph Pad Prism (version 3.02). The inset shows mean EC50±SEM, n = 3 independent experiments; EC50 are not significantly different (p>0.05).

To further analyze *Bv*PIP2;1 in terms of its capacity to modulate water permeation under cytosolic acidification, we performed swelling assays under different acidic (intracellular) conditions. Figure 4 shows that the pattern of P*_f_ versus* pH is sigmoidal, suggesting that *Bv*PIP2;1 could be an allosteric channel. The characterizing parameters of *Bv*PIP2;1 pH response are a EC_50_ equal to 6.41±0.05 and a P*_f_* maximal inhibition of (88±3)% at pH_int_ 6.3 (media ± SEM, n = 3 independent experiments). The reduction of P*_f_* suffered by the oocyte membranes expressing *Bv*PIP2;1 at acidic pH is not partial as the previously reported for the expression of other PIP2. For instance, *Bv*PIP2;2 and *Fa*PIP2;1 show a partial inhibition at acidic pH of 70% and 52% respectively [17], [26].

When the pH effect is tested on oocytes co-injected with cRNA encoding for *Bv*PIP2;1 and *Bv*PIP1;1, the maximal inhibitory response found was (87±4)%, which means that there is no significant difference with the inhibition found for *Bv*PIP2;1 alone (Figure 4). Moreover, the EC50 of the dose-response curve of oocytes injected only with cRNA of *Bv*PIP2;1 in comparison with the EC50 corresponding to curves of oocytes co-injected with *Bv*PIP2;1 and *Bv*PIP1;1 (EC_50_ equal to 6.47±0.01) are also not significantly different (p>0.05) (Figure 4). This result reinforces the hypothesis that *Bv*PIP2;1 does not promote the incorporation of *Bv*PIP1;1 in oocytes plasma membrane and that the inhibition measured corresponds to the blockage of *Bv*PIP2;1 alone.

### *Bv*PIP2;1 Loop A

**Figure 5:**
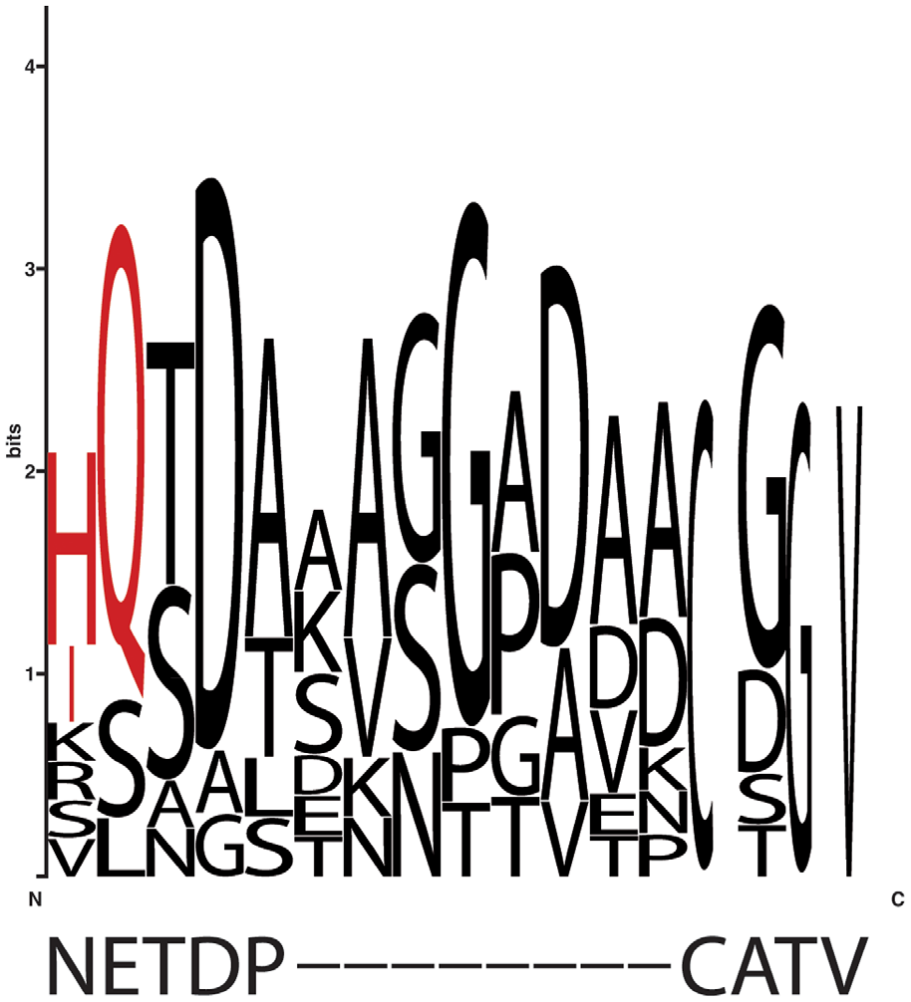
Conserved residues of reported PIP2 that interact with PIP1. Alignment of the PIP consensus sequences between the last aminoacid of the first TMH-transmembrane helix- and the first residue of the second TMH, including loop A (residues 64–72). The PIP consensus sequence is based on PIP2 that interact with PIP1. Sequence conservation is displayed by the sequence logos technique. The corresponding residues in *Bv*PIP2;1 primary sequence, according to this alignment, are shown down the logo. In red are indicated the selected residues to be mutated.

Recently, PIP loop A has been described as important in the stabilization of PIP dimer formation [19]. Here, we focus our study on the characteristics of *Bv*PIP2;1 loop A. First, we found some differences in comparison with the primary sequence of other PIP2 previously reported to be able to interact with PIP1. *Bv*PIP2;1, as other PIP belonging to PIP2, present a gap in their loop A and two low frequency residues, N64 and E65 (Figure 5).

It is unknown if the gap found in loop A is relevant for PIP2-PIP1 functional interaction. Some PIP2 with a short loop A have been reported to functionally interact with some PIP1, as can be the case of *Vv*PIP2;2-*Vv*PIP1;1 [14] and *Nt*PIP2;1-*Nt*PIP1;1 [12]. Since the findings of PIP2-PIP1 interaction are still singular reports, a generalization about the relevance of the gap in some PIP2 cannot be stated. On the other hand, the concernment of N64 and E65 in the lack of functional interaction found for *Bv*PIP2;1 and *Bv*PIP1;1 will be analyzed in the next section (See *Bv*PIP2;1 mutants).

In order to explore the structure and dynamics of *Bv*PIP2;1 loop A we built an homology model (HM) based on the chain A of *So*PIP2;1 crystal and performed MDS. After HM observation, it was seen that loop A is defined by residues from position 65 to 72 of the primary sequence and is oriented towards the center of the tetramer. Interestingly, the residues N64 and E65 are the last residue of the first alpha helix and the first residue of the loop A, respectively.

**Figure 6:**
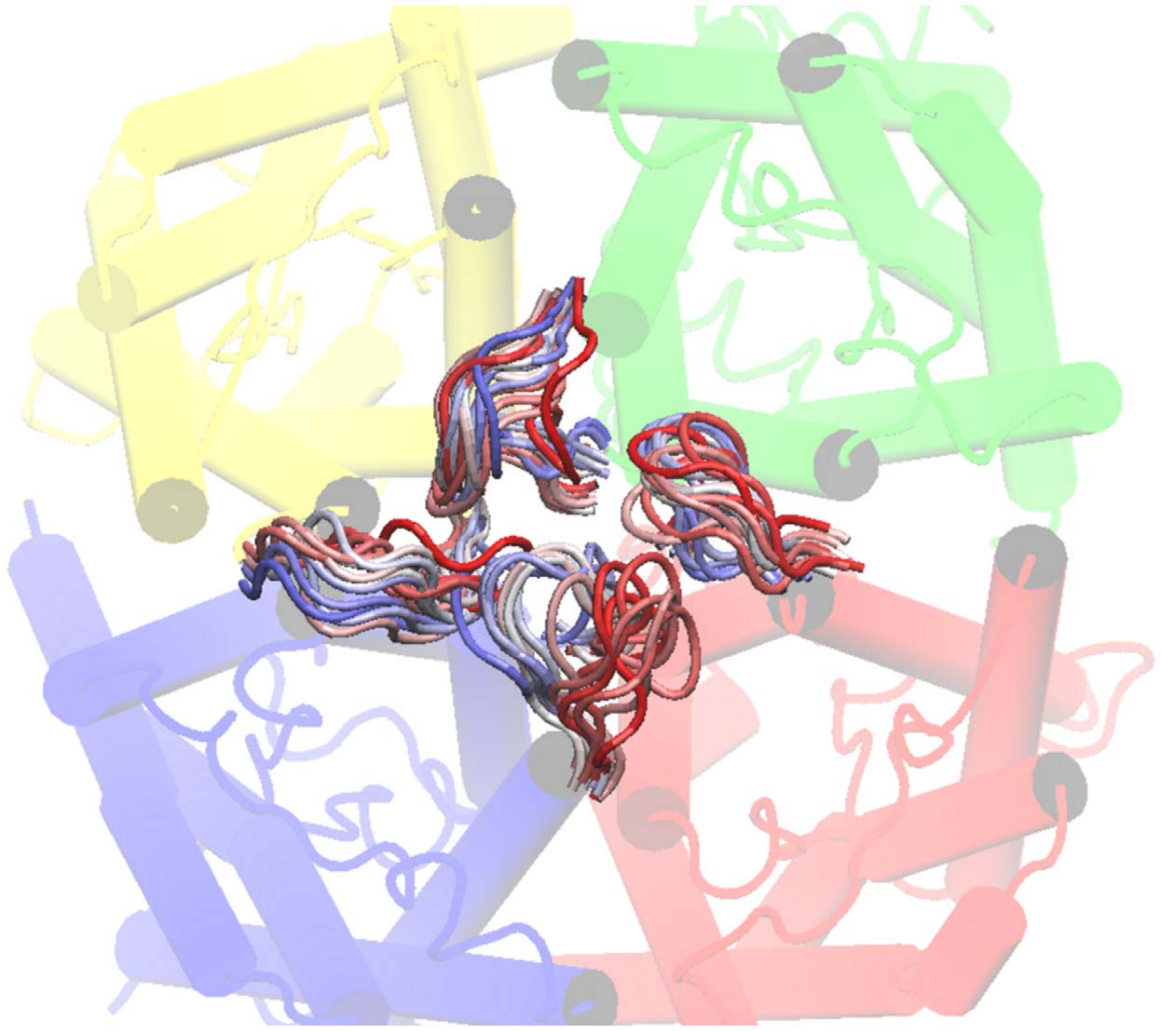
*Bv*PIP2;1 loops A MDS frames superimposition. The figure shows the MDS for *Bv*PIP2;1 loops A. Each *Bv*PIP2;1 monomer is in a different color; chain A is in yellow, chain B is in green, chain C is in blue and chain D is in red. The superimposition of 30 ns MDS of *Bv*PIP2;1 loops A is shown in a color range, where red is the starting position, white is an intermediate position and blue is the final one.

**Figure 7:**
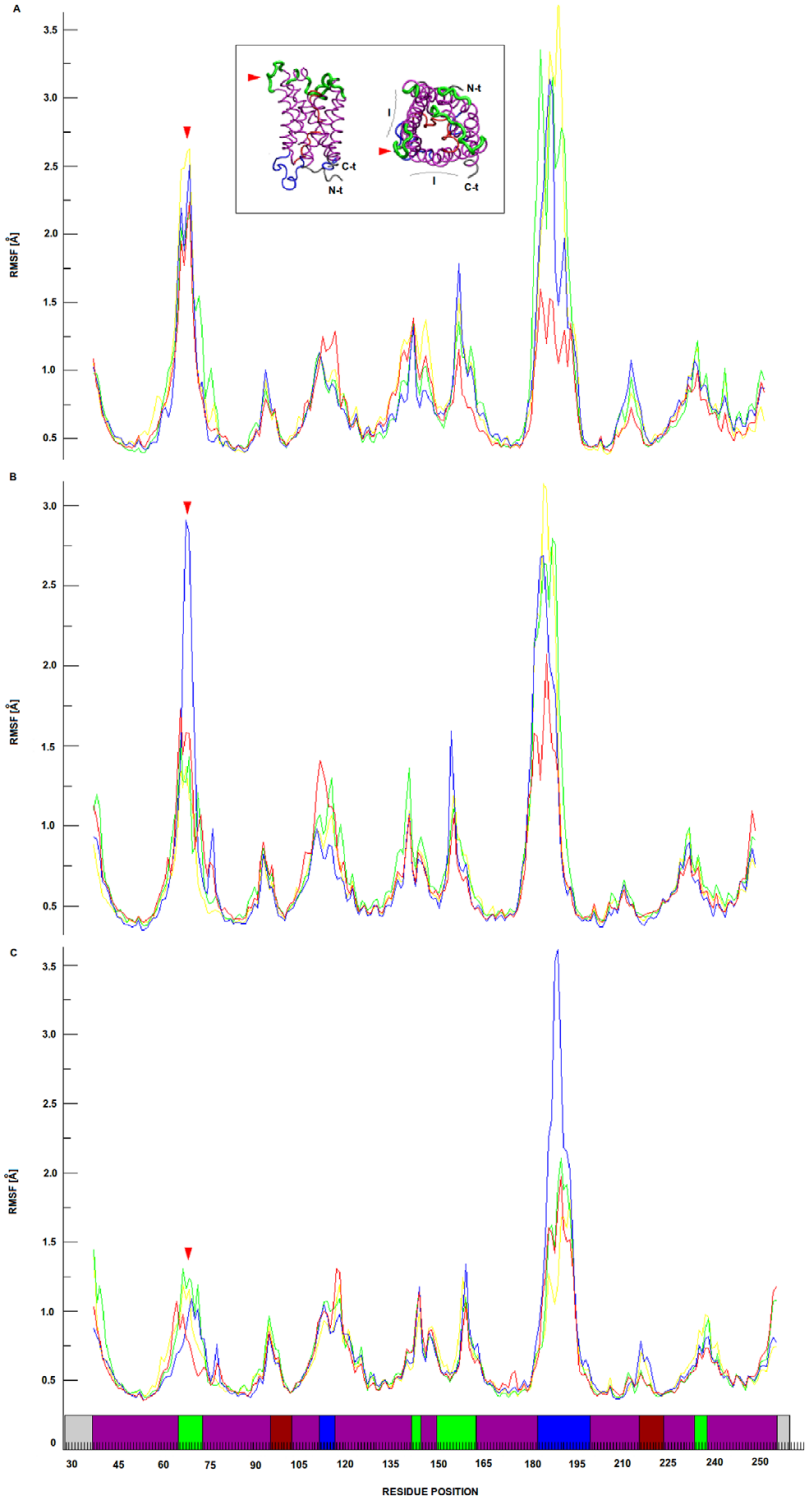
*Bv*PIP2;1 loop A root mean square fluctuation (RMSF). The RMSF of loops A are shown in yellow, green, blue and red lines corresponding to chains A, B, C and D respectively in all panels. Panels from A to C represent temporal intervals of the MDS ranging from 0 to 10 ns, 10 to 20 ns and 20 to 30 ns respectively. The inset shows the *Bv*PIP2;1 model, where green is used to point extracellular elements of the aquaporin, blue to point intracellular elements, purple to mark alpha helixes, bordeaux to distinguish loops B and E embedded in the membrane region and finally grey to show N-t and C-t. The same color pattern is used in the x-axis of panels to discriminate the location of residues of the primary structure in the protein. In the inset a red arrow is used to point loop A. The figure shows valleys in the RMSF which points that the secondary structure remains stable, the peaks represent movable parts of the protein and comparing extracellular elements, loop A is most flexible than loop C along the whole MDS.

The MDS showed that each of the four loops A in the tetramer developed a non-equivalent movement, since when starting in identical positions they reached different final conformations after 30 ns of simulation (Figure 6). Regarding the putative fluctuation of the four monomeric loop A positions, RMSF shows that these loops are the most flexible elements of the external face of each monomeric *Bv*PIP2;1 along the whole MDS (Figure 7). Our results also confirm that loops D are the most flexible parts of the inner face of the channel in accordance with functional features proposed for this loop [5], [29], [33].

MDS points out that the loops A of some monomers could be in contact; indeed the analysis of the distances of all possible pairs of loop A Cys residues (Figure S4) suggest the possibility of S-S bridges formation between contiguous monomers in accordance with previous works [19], [34].

A further characterization of loop A behavior in each monomer was assessed by root mean square deviation (RMSD), radial distribution function (g(r)) and Ramachandran plot. RMSD of each loop was calculated along the whole MDS. The result obtained reinforced that loop A belonging to a particular monomer has different movements and visits different conformational spaces along the whole MDS since the dynamic convergence is reached in their particular ways (Figure S5).

The g(r) functions are in agreement with full hydration of loop A aminoacids polar groups since in all cases a peak corresponding to the first solvation sphere is observed (Figure S6). It is worth noting that some identical polar groups in each monomer show different solvation patterns, as is the case of T66, C69 and T71. On the other hand, the atoms of D67 residues of the four chains have very similar solvation patterns may be due to the distance of this residue from the anchoring extremes of the loop. Considering loop A orientation, this high and even differential exposure of some residues in the loops A may be important to facilitate inter-monomer interactions within the tetramer.

Lastly, Ramachandran plots of each loop A residues show that the backbone conformational space is differently explored for some particular residues depending on the monomer they belong to (Figure S7), enhancing the idea of four loops A having different movements.

All these results together point to the possibility of loops A mediating contacts between monomers and let us speculate that aminoacid substitutions may have functional significance.

### *Bv*PIP2;1 Mutants

The above mentioned two non-conserved residues N64 and E65 resulted interesting due to their location: N64 is the last residue of the first transmembrane helix and E65 is the first residue of loop A. So, these residues may play a role in the flexibility of the loop. These characteristics make those residues attractive to be mutated. Considering the conserved residues in other PIP2 with reported interaction with PIP1 (Figure 5), we replace N64 and E65 of *Bv*PIP2;1 in order to obtain the following mutants: *Bv*PIP2;1 N64H/E65Q and *Bv*PIP2;1 N64I/E65Q.

**Figure 8:**
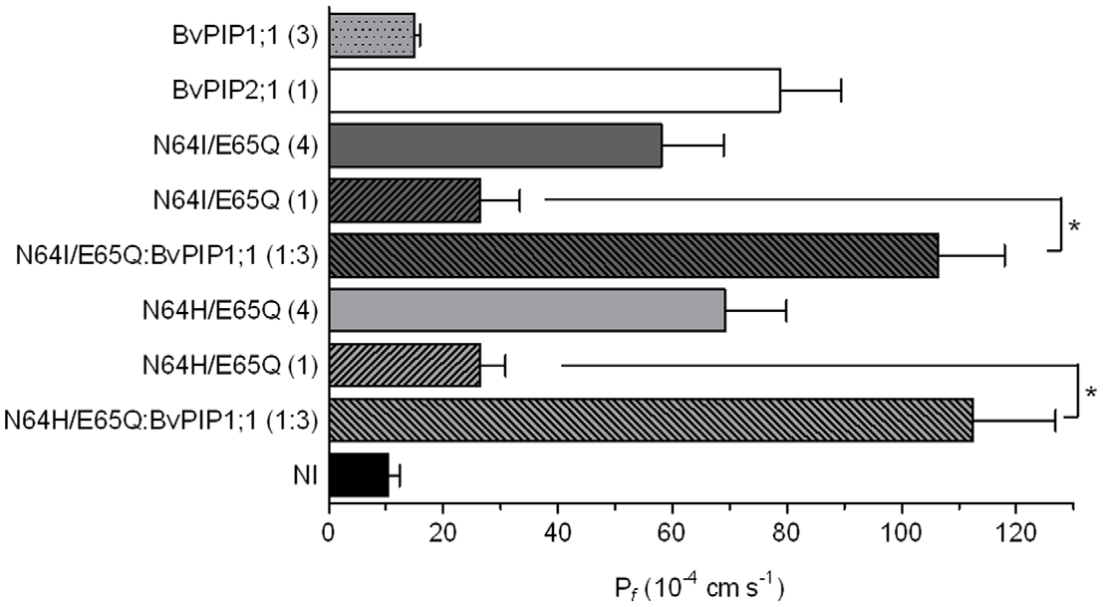
Osmotic permeability (P*_f_*) of oocytes membranes expressing *Bv*PIP2;1 mutants (N64H/E65Q or N64I/E65Q) with *Bv*PIP1;1. *Bv*PIP2;1 mutants (N64H/E65Q and N64I/E65Q) show high water transport activity indicating that they are functional aquaporins. Both mutants are able to functionally interact with *Bv*PIP1;1 as they generate oocyte membrane P*_f_* values far superior to those promoted by the mutant alone (*p<0.001). NI are non-injected oocytes. Values are representative data of three independent experiments using different oocyte batches. For each condition mean values are shown as mean P*_f_* ±SEM, n = 12−15.

When expressed in Xenopus oocytes the two mutants give rise to a lower P*_f_* than the developed by *Bv*PIP2;1 expression, notwithstanding they promote a significant increase in oocyte water permeability showing they are functional aquaporins (Figure 8). Interestingly, the co-injection of cRNA encoding *Bv*PIP1;1 and *Bv*PIP2;1 N64I/E65Q triggered the membrane P*_f_* to a value 4.1 times higher than the one promoted by the mutant *Bv*PIP2;1 N64I/E65Q alone. A similar result was found for the other mutant, as the co-injection of cRNA encoding *Bv*PIP1;1 and *Bv*PIP2;1 N64H/E65Q results in an 4.3 fold increase of P*_f_*. In contrast with *Bv*PIP2;1, both mutants seem to functionally interact with *Bv*PIP1;1 (Figure 8).

**Figure 9:**
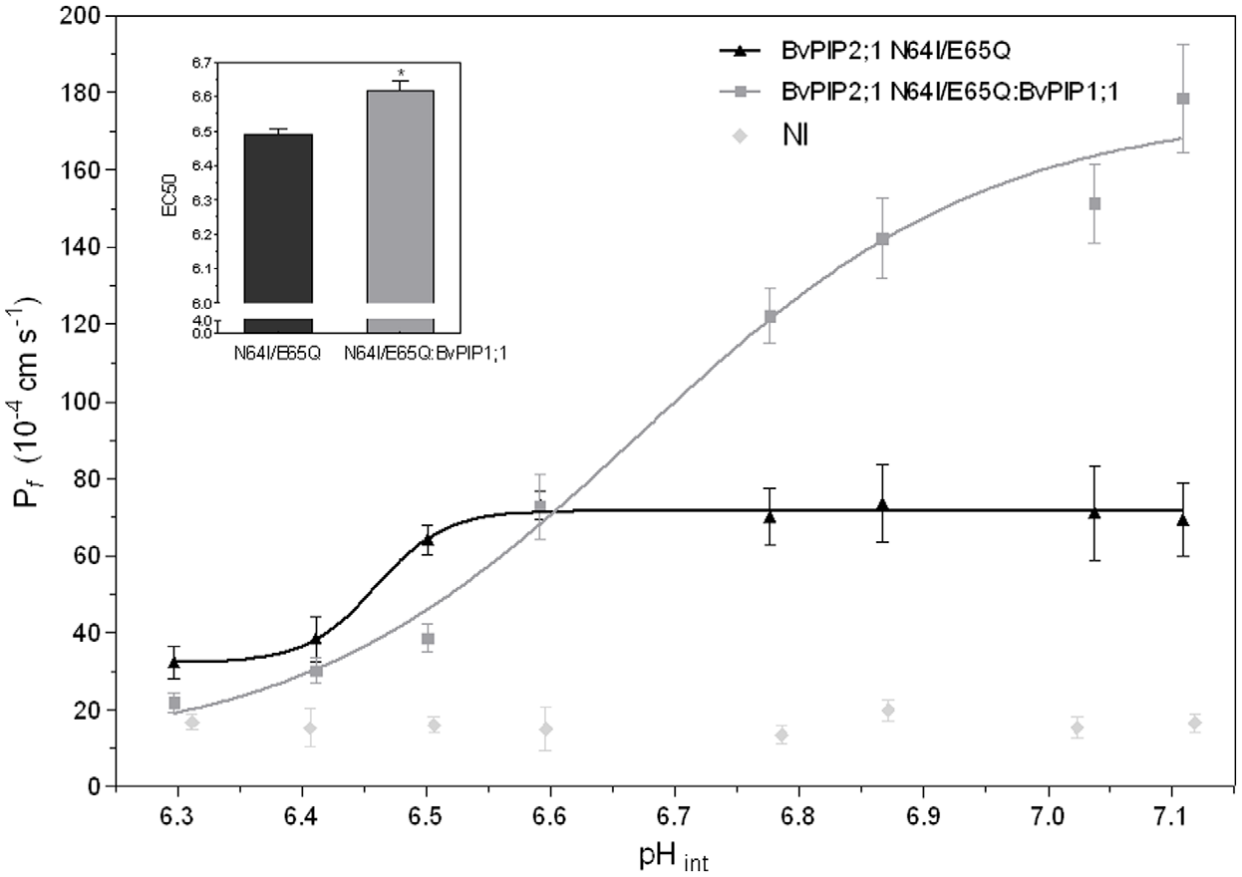
pH dose-response curve of *Bv*PIP2;1 N64I/E65Q mutant and its co-injection with *Bv*PIP1;1. The effect of cytosolic acidification on P*_f_* was tested on oocytes injected with *Bv*PIP2;1 N64I/E65Q cRNA alone or co-injected with *Bv*PIP1;1 cRNA in a 1∶1 mass ratio. Co-expression of *Bv*PIP2;1 N64I/E65Q:*Bv*PIP1;1 account for a different pH sensitivity in comparison with *Bv*PIP2;1 N64I/E65Q alone. In the main figure values are representative data from three independent experiments using different oocyte batches. For each condition mean values are shown as P*_f_* ± SEM, n = 7−10. Data were fitted to a sigmoidal dose-response curve using Graph Pad Prism (version 3.02). The inset shows mean EC50±SEM, n = 3 independent experiments, EC50 are significantly different (*p<0.05).

When the activity of the mutant under acidification was assessed we found that even when the response profile of *Bv*PIP2;1 N64I/E65Q expressed alone or co-expressed with *Bv*PIP1;1 remained both sigmoidal, their parameters are different: while *Bv*PIP2;1 N64I/E65Q presents a partial pH maximal inhibition at pH_int_ 6.3 and a EC50 equal to 6.49±0.02 (media ± SEM, n = 3 independent experiments), the co-expression suffered a total blockade at the same pH_int_ and the EC50 was 6.61±0.03 (media ± SEM, n = 3 independent experiments) (Figure 9). This strong modification of pH response further support the idea of *Bv*PIP2;1 N64I/E65Q mutant functionally interacting with *Bv*PIP1;1.

## Discussion

*Bv*PIP1;1, *Bv*PIP2;1 and *Bv*PIP2;2 are the only three *Beta vulgaris* PIP aquaporins described and they have been found to be expressed in different red beet tissues [17]. It was previously shown that *Bv*PIP2;2 functionally interact with *Bv*PIP1;1 increasing oocytes membrane P*_f_* and modifying the pH response, from partial to complete shutdown, after cytosolic pH acidification [17].

In the present work we found that *Bv*PIP2;1 does not behave as *Bv*PIP2;2, neither concerning its functional interaction with *Bv*PIP1;1, nor about its inhibition under cytosolic acidification. With regard to this last feature we found that *Bv*PIP2;2 is partially blocked at pH_int_ 6.4 while *Bv*PIP2;1 is completely inhibited. Regarding *Bv*PIP2;1 ability to interact with a PIP1, the complete set of results presented in this work, i.e. water transport measurements and localization pattern studied by confocal fluorescence microscopy, shows that *Bv*PIP2;1 do not interact with *Bv*PIP1;1. These results are in accordance with previous findings on *Beta vulgaris* water channels which, by means of biochemical approaches, suggested that the PMIP31 and PMIP27 (now known as *Bv*PIP2;1 and *Bv*PIP1;1) do not form mixed heterodimeric species [34].

Aquaporin heterooligomerization is emerging as an important point of regulation and fine-tuning of water transport. Many evidences in the literature point to the existence of PIP aquaporins as oligomers formed by a combination of PIP1 and PIP2: i- increased water transport rates due to functional interaction between PIP1 and PIP2 channels have been detected in several plant species [11], ii- FRET studies show that PIP1 and PIP2 aquaporins are near enough to form heterooligomers [11], iii- pH_int_ sensing in co-expressions of PIP1 and PIP2 systems is different from pH_int_ sensing of PIP2 expressed alone [17], [26], iv- changes in transport selectivity have been reported as modified when heterotetramers of PIP1 and PIP2 are constructed [18], v- modification of PIP1 localization from ER to plasma membrane was detected only when PIP2 is also expressed in the same cell [11], [13].

Along with all this strong evidence, in the last years it has been widespread that PIP2 and PIP1 can form heterooligomeric assemblies. However, there are reports showing some PIP2 that do not functionally interact with PIP1 [11], [35], [36]. In this subset of plant water channels should be included *Bv*PIP2;1.

We proposed that *Bv*PIP2;1 loop A could be the key element for the lack of functional interaction with *Bv*PIP1;1 after *Bv*PIP2;1 primary sequence comparison with other PIP2, HM and MDS evaluation and the consideration of loops as relevant elements mediating protein-protein interaction [37], [38]. Indeed, Hayward and Kitaos [39] stresses the importance of fixed ends in the configuration of protein loops. The authors remark that this constraint might influence the dynamical behavior of the loop. Interestingly, when we mutated the C-terminal of *Bv*PIP2;1 helix 1 and the N-terminal of *Bv*PIP2;1 loop A for two residues highly conserved in PIP2 that have been reported as able to functionally interact with PIP1, i.e. H or I for position 64 and Q for position 65 (Figure 5), the mutated channels acquired the capacity to interact with *Bv*PIP1;1.

The functional interaction between *Bv*PIP2;1 mutants and *Bv*PIP1;1 is demonstrated by an enhanced of fourfold oocyte plasma membrane P*_f_* in comparison with *Bv*PIP2;1 mutants alone (Figure 8 and Figure 9) and also by a change in the inhibitory pH response pattern (Figure 9).

With regard to plant aquaporin loops, main attention was focused on intracellular loop D, which has been described as responsible for the gating of PIPs [5], [29], [33]. Also, loop A has previously received some consideration. For example, a highly conserved cysteine residue located in PIP loop A has been suggested as having a relevant function in tetrameric organization. It was reported that due to a particular configuration of *So*PIP2;1 loop A, the four cysteines (one in each monomer) may play a role in the stabilization of the tetramer since according to their nearness they can form hydrogen bonds or complex metal ions [40]. Moreover, in a recent work, it was shown that those cysteines could play a role in dimer stabilization of *Zm*PIPs [19].

When the overall architecture of *So*PIP2;1 was studied, it revealed considerable similarity to the mammal AQP1 [3], [41]. Notwithstanding, differences in the two first transmembrane helices render a different position of the connecting loop A [40]. In AQP1, loop A seems to have a position parallel with the side of the monomer, while in *So*PIP2;1 the positions of the C-terminus of helix 1 and N- terminus of helix 2 suggest that loop A is oriented towards the center of the tetramer [40]. In the present work, the MDS of *Bv*PIP2;1 shows that the position of loop A is also oriented towards the center of the tetramer (Figure 6) in accordance with results found for *So*PIP2;1. Furthermore, these results indicate that the four loops A are flexible parts of the monomers each having a different solvation pattern and different movement along the MDS.

Our functional experiments show the importance of loop A as involved in *Bv*PIP2;1 mutants and *Bv*PIP1;1 functional interaction. This result in combination with the MDS data about flexibility and orientation of loop A facing towards the center of the tetramer could be indicating a role of these loops as mediators in the interaction between contiguous monomers of a same tetramer. Despite up to date we cannot offer a thorough mechanism of loop A participation in monomer-monomer contact, our results are an evidence in favor of the plausibility of heterotetramerization. The mutation of two conserved residues of this loop could alter its charge or steric hindrance allowing or preventing PIP1-PIP2 heterotretramers formation.

Resuming, *Beta vulgaris* aquaporins present a specific pattern of activity and functional interaction, where *Bv*PIP2;1 is organized as homooligomers (due to, at least, its loop A structure) while *Bv*PIP2;2 and *Bv*PIP1;1 are able to assemble as heterooligomers.

In previous works we studied the water transport characteristics of *Beta vulgaris* plasma membrane vesicles (PMV) [42], [43]. We reported that, under acidification, water transport through PMV was completely shut down. So, all water channels present in those PMV should be completely inhibited at low pH. This result obtained on plant material is consistent with the presence of *Bv*PIP2;1 homotetramers and *Bv*PIP2;2-*Bv*PIP1;1 heterooligomers responding in a concerted way when pH drops to acidic values.

As a general consideration, we would like to stress that the assembly of water channels as heterooligomers is frequently studied by straightforward functional assessment, generally by means of cRNA co-injection of different PIP isoforms in an heterologous system like *Xenopus* oocytes followed by water transport measurements, i.e. P*_f_* determination. Many results show that P*_f_* of oocytes co-expressing PIP1 and PIP2 is higher than the obtained for PIP2 expression alone. However, on the basis of published results, the increment of P*_f_* does not always happen at the same extent and the threshold of P*_f_* representing an authentic PIP interaction is not well established. At this respect some PIP1 interacting with PIP2 triggered an increase in the membrane P*_f_* of seven fold [17], four fold [26] or even three fold [14], while other PIP2 interacting with PIP1 only leads to a 1.4 fold increment [15]. Additionally, the increase in the membrane P*_f_* sometimes seems to be dependent of the ratio of injected PIP1:PIP2 cRNA in the oocyte [11], [12], while in other cases seems to be independent [14]. Under this complex scenario we suggest that, to further confirm that a PIP2 is interacting with a PIP1, it becomes important to take into account different experimental approaches, not only the increment of membrane P*_f_*. It can be considered, together with water transport assays, the localization pattern of the different PIP isoforms studied by confocal fluorescence microscopy or the pH inhibitory response observed after oocyte cytosolic pH acidification changes when the plasma membrane expresses only one type of PIP in a homotetrameric way or a mix of two different PIP. Therefore, a combination of experimental approaches could overcome the uncertainty about a genuine PIP2-PIP1 interaction that could arise from the lack of a confident cut off value for water transport increases when comparing P*_f_* resulting from PIP2 expressing systems with PIP2-PIP1 co-expressing ones.

In summary, in this work we found that: i- not all PIP2 are able to interact with any PIP1, since at least *Bv*PIP2;1 is unable to promote the incorporation of *Bv*PIP1;1 in the plasma membrane, and ii- loop A is relevant for PIP1-PIP2 functional interaction since mutations in this loop modify the behavior of *Bv*PIP2;1-*Bv*PIP1;1 co-expression.

The puzzle of PIP aquaporins interaction and localization is being unveiled in these last years, however despite all this detailed information, the structural elements that can be involved in the formation of heterooligomeric, or heterotetrameric, assemblies are still under investigation. Our results broaden previous findings on the significant role of loop A in monomer-monomer contacts in PIP aquaporins showing that loop A not only may play a role in stabilizing contacts among monomers, but also can be a controller of heterooligomerization.

## Supporting Information

Figure S1 **Phylogeny of PIPs2 in plants.** Phylogenetic trees of PIPCLII and PIPCLIII protein sequences from representative taxa based on NJ method are shown. Bootstrap percentages are indicated at the branch points. Orthologous gene clusters (CL) are found on right. Tree topology obtained using NJ method, Minimum evolution and Maximum parsimony methods were identical.
(TIF)

Figure S2 **Phylogeny of PIPs1 in plants.** Phylogenetic tree of PIPCLI protein sequences from representative taxa based on NJ method is shown. Bootstrap percentages are indicated at the branch points. Tree topology obtained using NJ method, Minimum evolution and Maximum parsimony methods were identical.
(TIF)

Figure S3 **Osmotic permeability (P** ***_f_*** **) of oocytes membranes expressing fluorescent tagged-PIPs.** All fluorescent-tagged *Bv*PIPs and BvPIP1;1-ECFP co-expressions show similar water transport activity than their corresponding wild types, indicating that the fluorescent tag do not modify their activities or functional interaction. NI are non-injected oocytes. Values are representative data of three independent experiments using different oocyte batches. For each condition mean values are shown as mean P*_f_* ±SEM, n = 7−10.
(TIF)

Figure S4 **Putative S-S bridges formed along the MDS between different loops A in the tetramer.** The figure shows the distances among all the possible pairs of sulfur atoms, corresponding to conserved cysteins residues in loop A, that might be involved in S-S bridges among the four chains along the MDS. The inset on top of the right establishes the color references for each pair of sulfur atoms (that correspond to A, B, C or D chains) shown in the figure. The inset on top of the left indicates a particular frame of the model where loops A are highlighted and yellow spheres (in CPK style) represent sulfur atoms. The inset in the center shows a schematic representation of the S-S bridges, between Cys of different monomers that can be formed at least during certain time window of the MDS.
(TIF)

Figure S5 ***Bv*** **PIP2;1 loops A root mean square deviation (RMSD).** The figure shows the RMSD for *Bv*PIP2;1 loops A calculated for the 30 ns of the MDS. The RMSD for each monomeric loop A is shown in a different color: loop A corresponding to chain A, B, C and D are in yellow, green, blue and red, respectively. It can be observed that a rather stable conformation is reached for the four loops at different times.
(TIF)

Figure S6 ***Bv*** **PIP2;1 loop A polar residues radial distribution function (g(r)).** The radial distribution function for each polar residue of *Bv*PIP2;1 loops A is shown up to 7 angstroms from the atomic position. In yellow, green, blue and red are represented the monomeric *Bv*PIP2;1 A, B, C and D chains respectively. Panel A and B correspond to the oxygen/and/of E65 residue, panel C to oxygen/of T66, panel D and E to oxygen/and/of D67, panel F to sulphur/of C69 and panel G to oxygen/of T71. In each panel it can be observed a peak that corresponds to the first solvation sphere for each atom; different solvation patterns are found in panels C, F, G.
(TIF)

Figure S7 **Ramachandran plot of** ***Bv*** **PIP2;1 loop A residues.** Ramachandran plots for each residue of *Bv*PIP2;1 loops A are shown. Loop A of chains A, B, C, D are organized in consecutive rows distinguished by yellow, green, blue and red labels respectively. As can be seen residues E65, T66, C69, A70, T71 and V72 explore the backbone conformational space in different ways depending on the chain they are part of, revealing that loops A are non-equivalent in *Bv*PIP2;1 tetramer.
(TIF)
