# Supplementary figures and images for: Loop A Is Critical for the Functional Interaction of Two Beta vulgaris PIP Aquaporins
Source: PLoS One. 2013 Mar 4;8(3):e57993. doi: 10.1371/journal.pone.0057993 (PMC3587573; doi:10.1371/journal.pone.0057993)

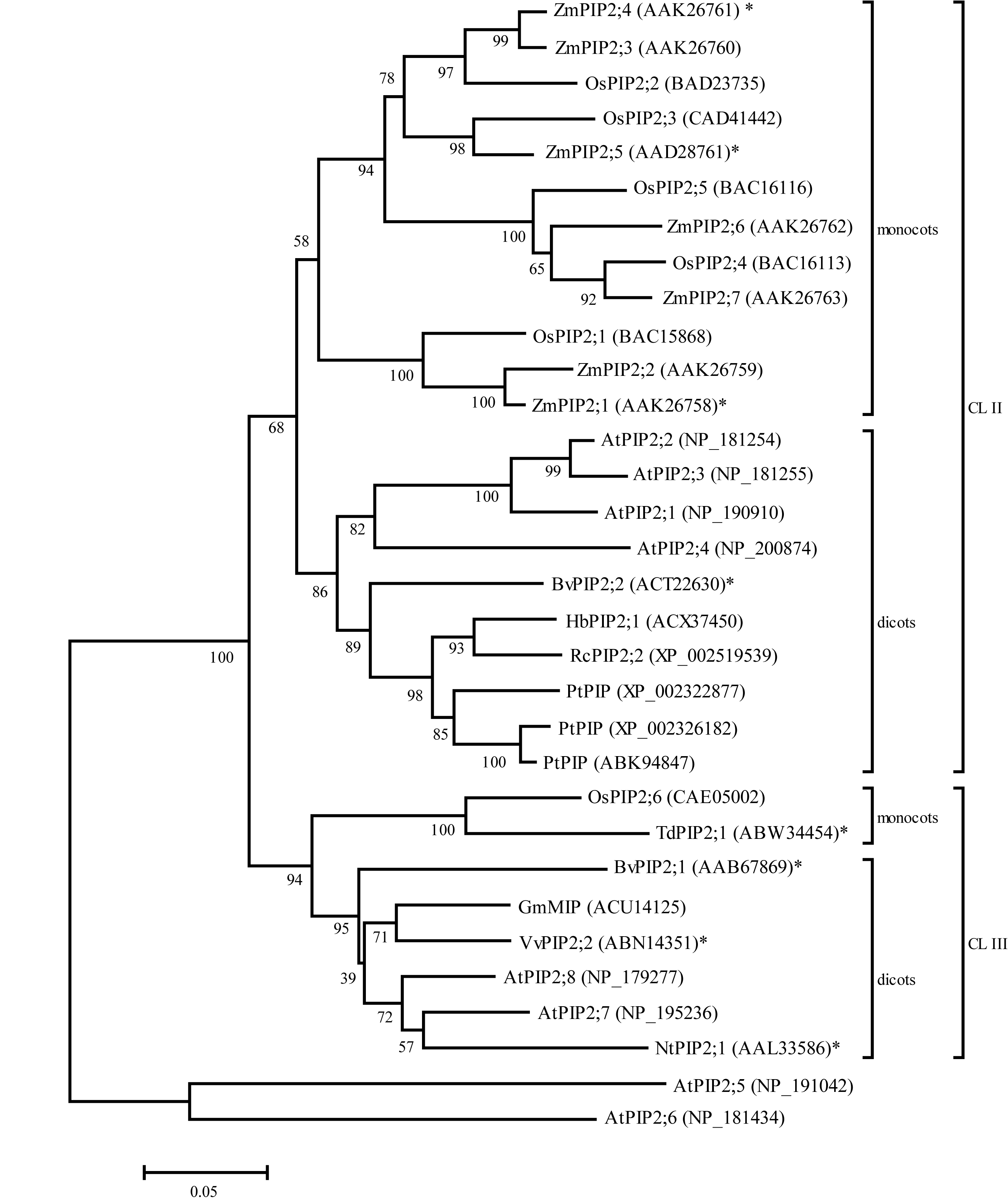

Supplement: Figure S1 — Phylogeny of PIPs2 in plants. Phylogenetic trees of PIPCLII and PIPCLIII protein sequences from representative taxa based on NJ method are shown. Bootstrap percentages are indicated at the branch points. Orthologous gene clusters (CL) are found on right. Tree topology obtained using NJ method, Minimum evolution and Maximum parsimony methods were identical. (TIF) [file pone.0057993.s001.tif]

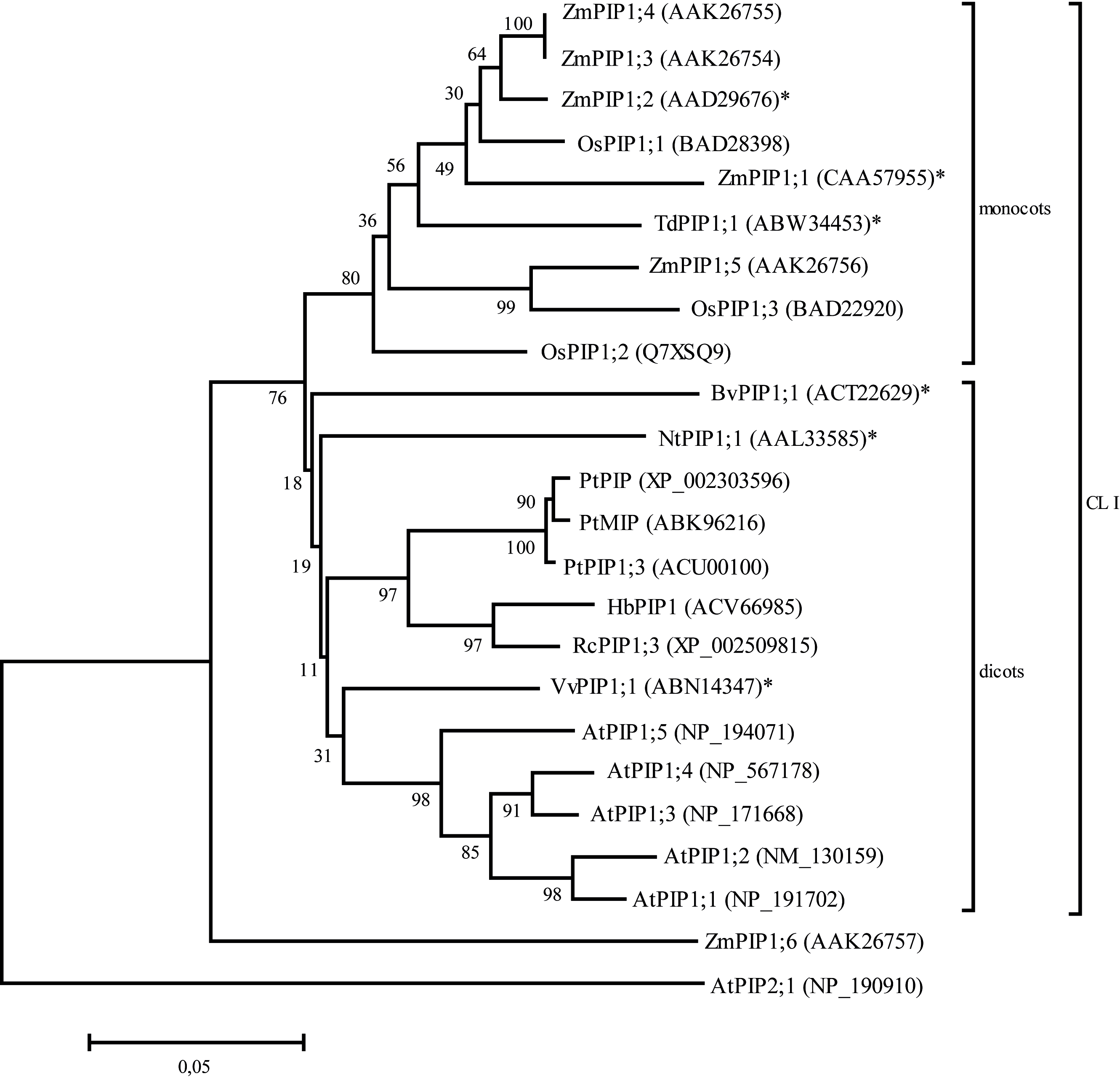

Supplement: Figure S2 — Phylogeny of PIPs1 in plants. Phylogenetic tree of PIPCLI protein sequences from representative taxa based on NJ method is shown. Bootstrap percentages are indicated at the branch points. Tree topology obtained using NJ method, Minimum evolution and Maximum parsimony methods were identical. (TIF) [file pone.0057993.s002.tif]

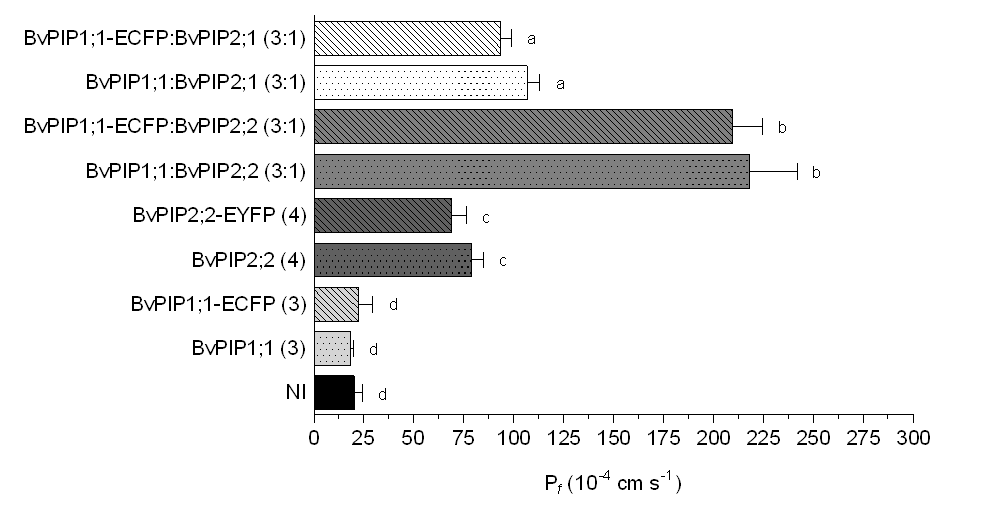

Supplement: Figure S3 — Osmotic permeability (P f ) of oocytes membranes expressing fluorescent tagged-PIPs. All fluorescent-tagged BvPIPs and BvPIP1;1-ECFP co-expressions show similar water transport activity than their corresponding wild types, indicating that the fluorescent tag do not modify their activities or functional interaction. NI are non-injected oocytes. Values are representative data of three independent experiments using different oocyte batches. For each condition mean values are shown as mean Pf ±SEM, n = 7−10. (TIF) [file pone.0057993.s003.tif]

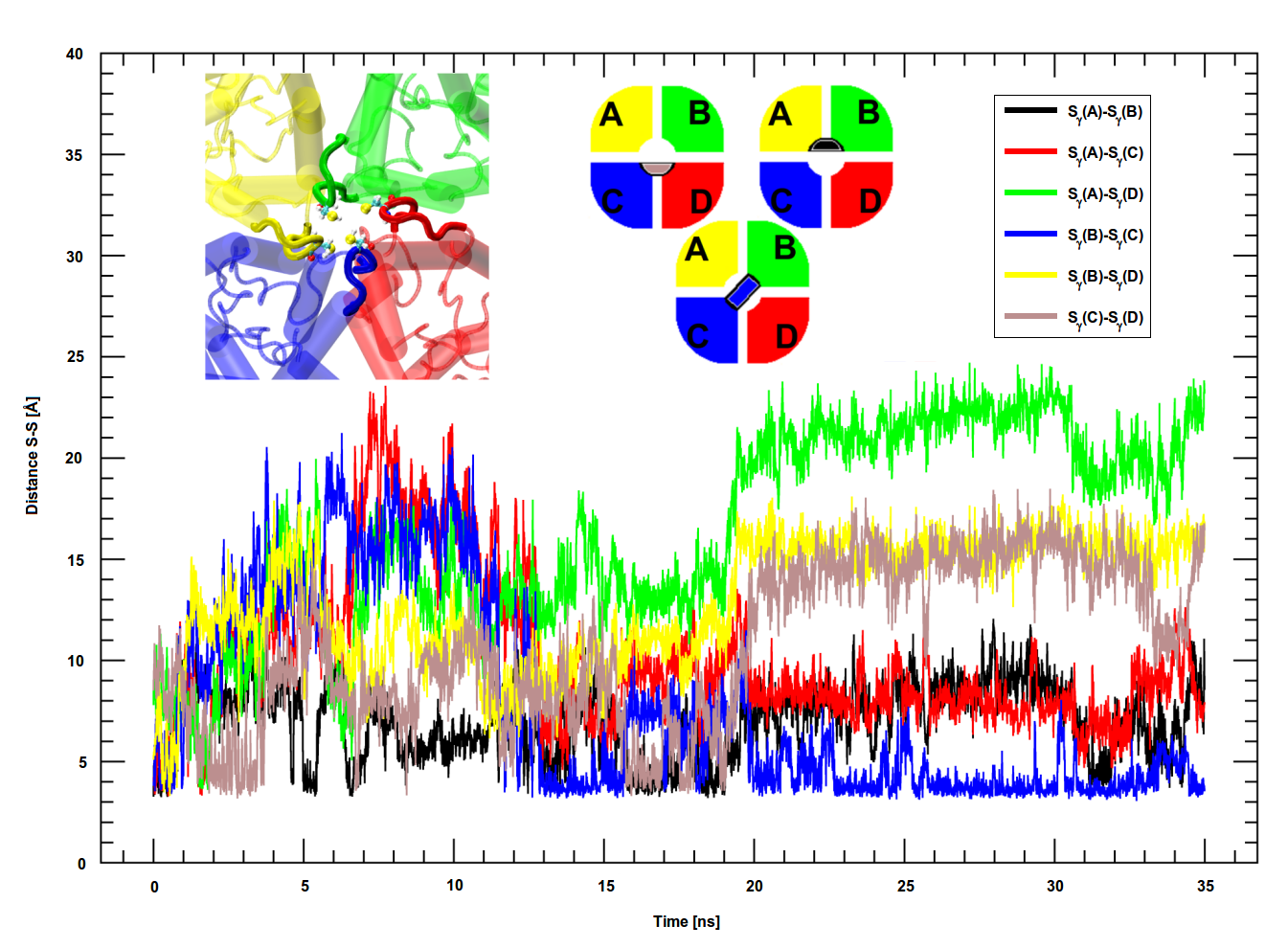

Supplement: Figure S4 — Putative S-S bridges formed along the MDS between different loops A in the tetramer. The figure shows the distances among all the possible pairs of sulfur atoms, corresponding to conserved cysteins residues in loop A, that might be involved in S-S bridges among the four chains along the MDS. The inset on top of the right establishes the color references for each pair of sulfur atoms (that correspond to A, B, C or D chains) shown in the figure. The inset on top of the left indicates a particular frame of the model where loops A are highlighted and yellow spheres (in CPK style) represent sulfur atoms. The inset in the center shows a schematic representation of the S-S bridges, between Cys of different monomers that can be formed at least during certain time window of the MDS. (TIF) [file pone.0057993.s004.tif]

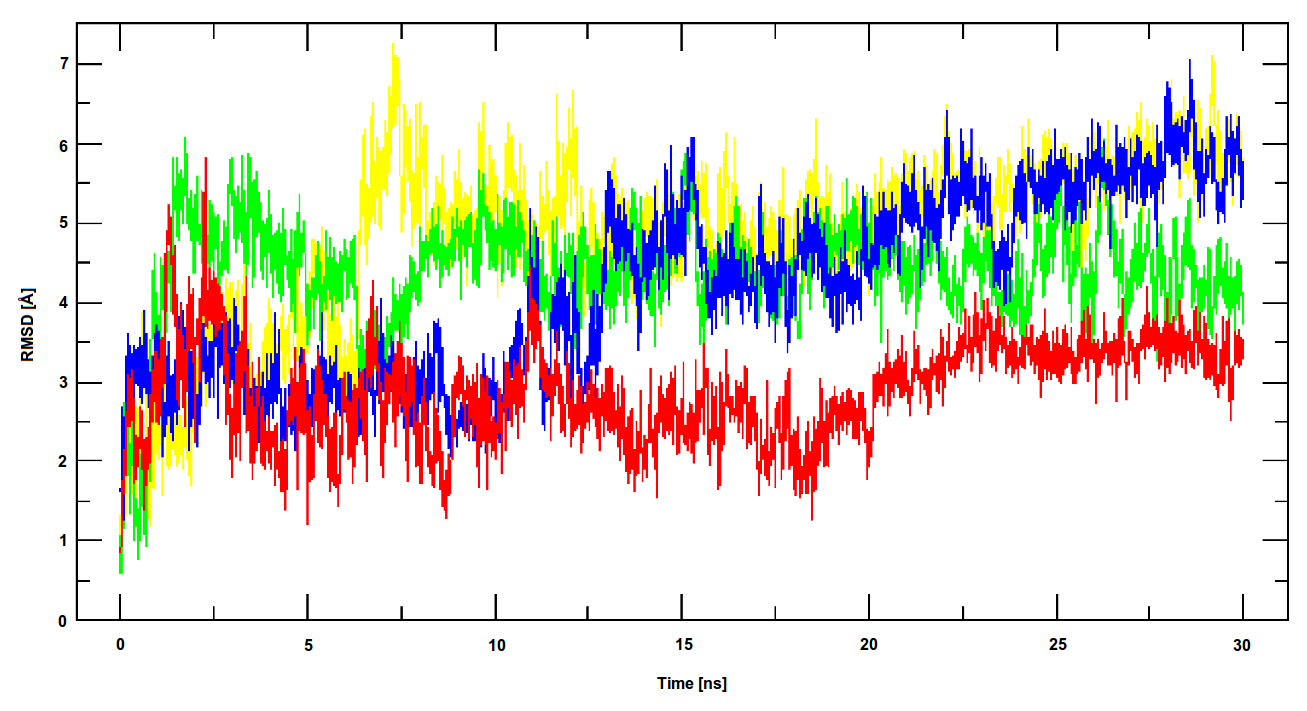

Supplement: Figure S5 — Bv PIP2;1 loops A root mean square deviation (RMSD). The figure shows the RMSD for BvPIP2;1 loops A calculated for the 30 ns of the MDS. The RMSD for each monomeric loop A is shown in a different color: loop A corresponding to chain A, B, C and D are in yellow, green, blue and red, respectively. It can be observed that a rather stable conformation is reached for the four loops at different times. (TIF) [file pone.0057993.s005.tif]

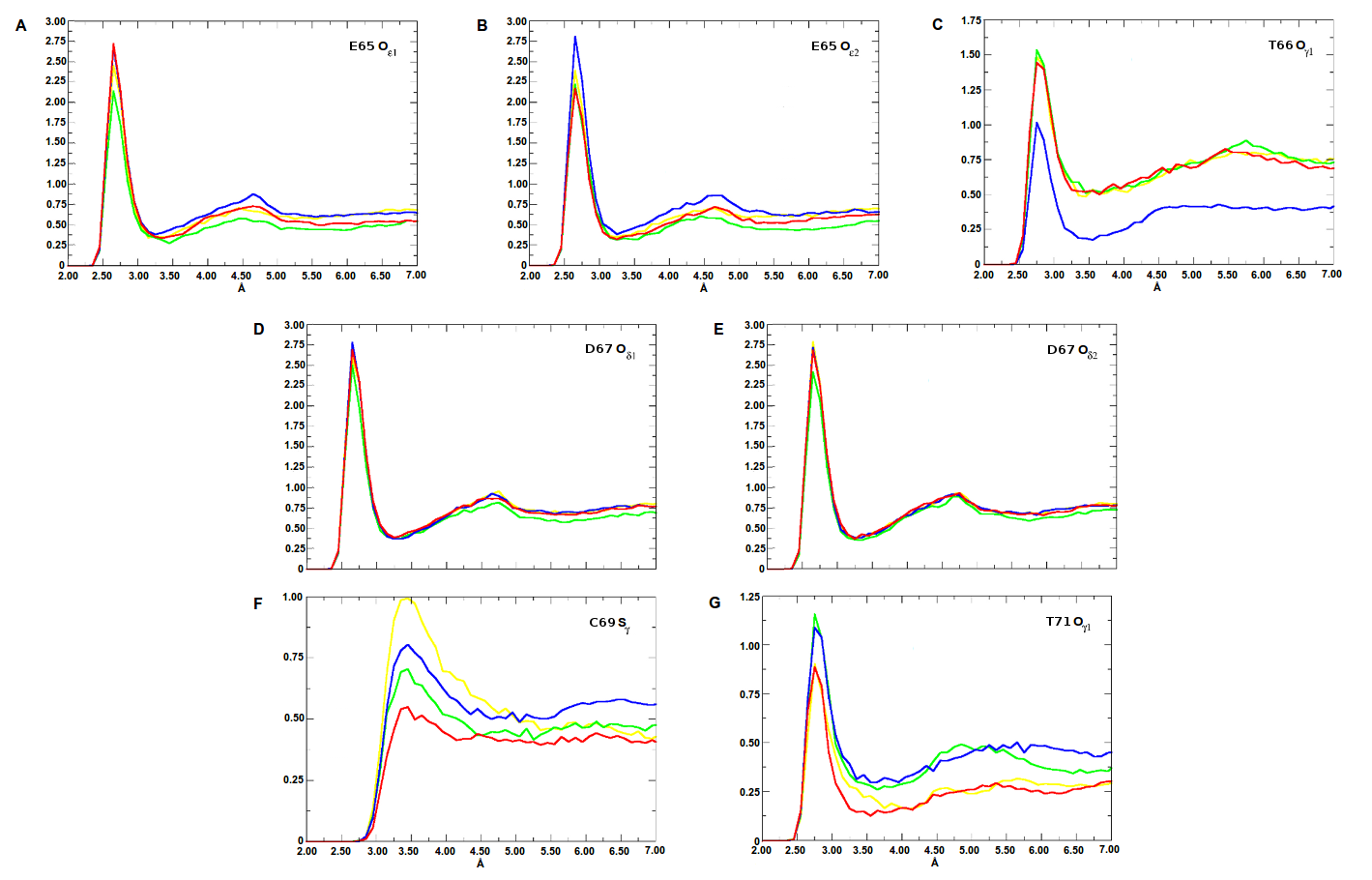

Supplement: Figure S6 — Bv PIP2;1 loop A polar residues radial distribution function (g(r)). The radial distribution function for each polar residue of BvPIP2;1 loops A is shown up to 7 angstroms from the atomic position. In yellow, green, blue and red are represented the monomeric BvPIP2;1 A, B, C and D chains respectively. Panel A and B correspond to the oxygen/and/of E65 residue, panel C to oxygen/of T66, panel D and E to oxygen/and/of D67, panel F to sulphur/of C69 and panel G to oxygen/of T71. In each panel it can be observed a peak that corresponds to the first solvation sphere for each atom; different solvation patterns are found in panels C, F, G. (TIF) [file pone.0057993.s006.tif]

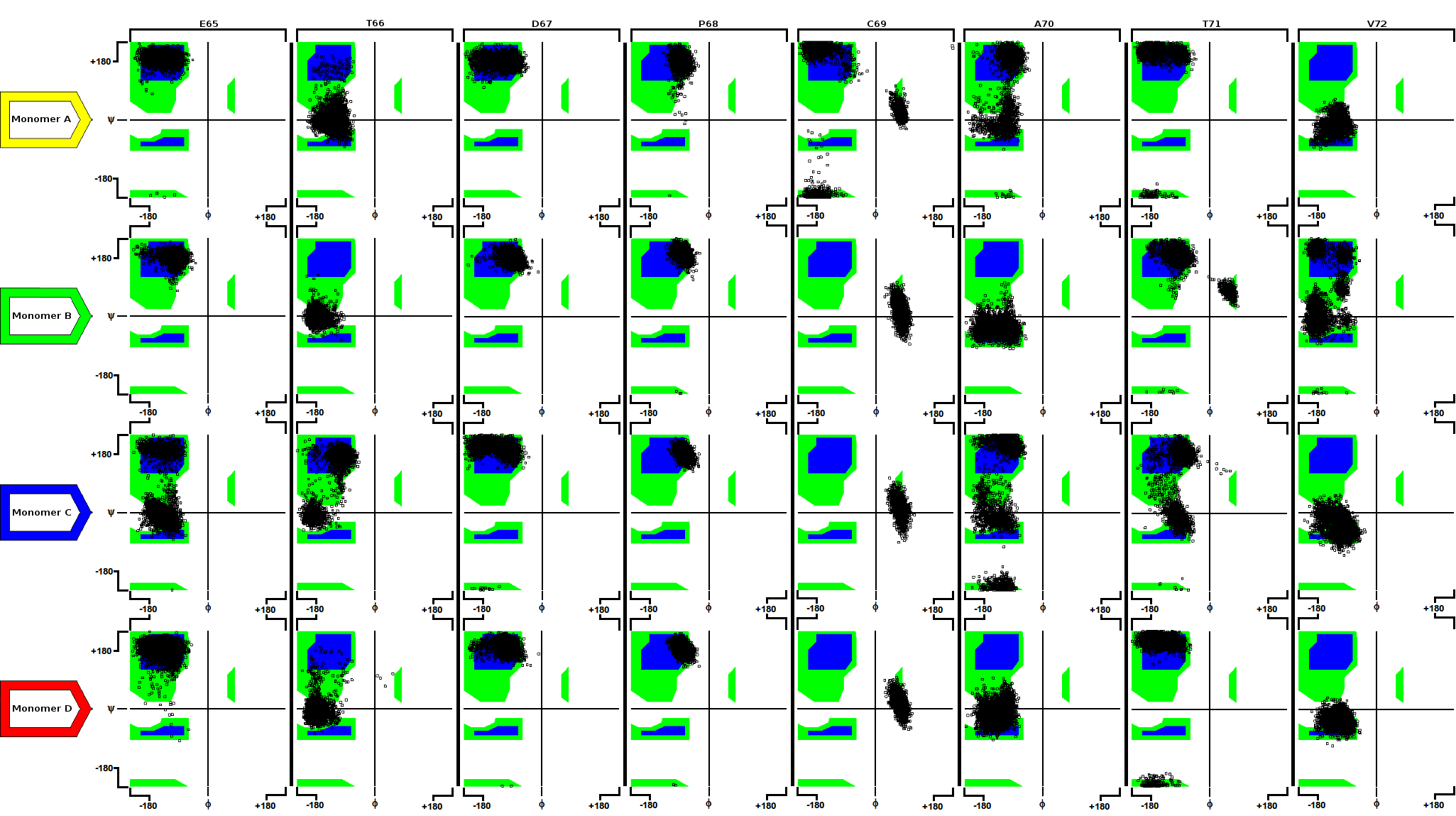

Supplement: Figure S7 — Ramachandran plot of Bv PIP2;1 loop A residues. Ramachandran plots for each residue of BvPIP2;1 loops A are shown. Loop A of chains A, B, C, D are organized in consecutive rows distinguished by yellow, green, blue and red labels respectively. As can be seen residues E65, T66, C69, A70, T71 and V72 explore the backbone conformational space in different ways depending on the chain they are part of, revealing that loops A are non-equivalent in BvPIP2;1 tetramer. (TIF) [file pone.0057993.s007.tif]
